# Genomic and Pathogenic Characterization of *Akanthomyces muscarius* Isolated from Living Mite Infesting Hazelnut Big Buds

**DOI:** 10.3390/genes15080993

**Published:** 2024-07-28

**Authors:** Silvia Turco, Mounira Inas Drais, Luca Rossini, Nicolò Di Sora, Federico Brugneti, Stefano Speranza, Mario Contarini, Angelo Mazzaglia

**Affiliations:** 1 Dipartimento di Scienze Agrarie e Forestali, Università degli Studi della Tuscia, 01100 Viterbo, Italy; drais@unitus.it (M.I.D.); nico.disora@unitus.it (N.D.S.); federico.brugneti@unitus.it (F.B.); speranza@unitus.it (S.S.); contarini@unitus.it (M.C.); angmazza@unitus.it (A.M.); 2 Service d’Automatique et d’Analyse des Systèmes, Université Libre de Bruxelles, 1050 Brussels, Belgium; 3 Centro de Estudios Parasitológicos y de Vectores (CEPAVE, CONICET-UNLP), La Plata B1900, Argentina

**Keywords:** fungal genomics, entomopathogen, de novo hybrid assembly, sequencing

## Abstract

The capability of entomopathogenic fungi to live as plant endophytes is well established. However, their presence in undiscovered environmental niches represents the beginning of a new challenging research journey. Recently, *Akanthomyces muscarius* (Ascomycota, Cordycipitaceae) (Petch) Spatafora, Kepler & B. Shrestha was isolated from hazelnut buds infested by the big bud mite pest *Phytoptus avellanae* Nalepa, which makes the buds swollen, reddish, and unable to further develop. Gall formation is known to be regulated by a consortium of microbes and mites, and to better understand the possible role of *A. muscarius* within the infested gall, its whole genome sequence was obtained using a hybrid approach of Illumina and Nanopore reads. The functional and comparative genomics analysis provided within this study may help answer questions related to the ecology and the entomopathogenicity of this fungus.

## 1. Introduction

The growing willingness to reduce pesticide use, strongly endorsed by international organisations and by several national and European regulations [1], is pushing the research of alternative methods to control arthropod pests and diseases. Several studies are currently focusing on natural enemies that have insect pests as main hosts, showing the strong potentiality of these methods [2,3,4]. Most of the studies are devoted to gaining information about the efficacy of predators, parasitoids, or entomopathogenic bacteria and fungi in controlling the populations of target species, highlighting the need for an in-depth knowledge of the biological, genetic, ecological, and behavioural aspects of the natural enemies involved.

Entomopathogenic fungi are among the most promising group of natural enemies that is arousing more interest from the scientific community, given their potential in plant protection. While their effects have been known for a long time [5], the advent of new technologies and investigation methods, such as genomics studies, is providing more accurate information on the interaction between entomopathogenic fungi and host insects.

To date, there are different isolates of fungi belonging to different orders and families and with different mechanisms of actions that may be potentially, or that are actually, commercialised for pest control purposes [5]. An example is the entomopathogenic fungus *A. muscarius* (Ascomycota, Cordycipitaceae) (Petch) Spatafora, Kepler & B. Shrestha, an isolate belonging to the family Cordycipitaceae, Hypocreales order, and previously known as *Verticillium lecanii*, *Lecanicillium lecanii*, or *L. muscarium* [6,7,8]. The genus *Akanthomyces* was proposed by Lebert (1858) to include entomopathogenic fungi infecting *Lepidoptera* and that are distinct from *Beauveria* and *Cordyceps* [9]. Nonetheless, *Akanthomyces* teleomorphs have been identified within the genera *Cordyceps* and *Torrubiella,* such as *C. confragosa* and *C. coccidioperitheciata* from spiders and scale insects, and *C. tuberculata* from adult moths [10]. Strains belonging to the *Akanthomyces* genus have been isolated from a plethora of organisms, including nematodes [11,12,13], insects and mites [14,15,16], and other plant-associated fungi [17]. *A. muscarius* is already known for its entomopathogenic activity on different groups of insects [18]. The main hosts belong to Hemiptera, especially Aleyrodidae, Aphididae, and Coccidae families [19,20], such as the economically relevant *Bemisia tabaci* (Gennadius) (Hemiptera: Aleyrodidae) [19], or different pest groups, as in the case of *Thaumetopoea pityocampa* (Denis & Schiffermüller) (Lepidoptera: Notodontidae) [21].

The mechanisms of action of *Akanthomyces* spp. consists of an initial adherence of the fungal conidia or blastospores to the host cuticle. Subsequently, hydrolytic enzymes such as proteases, chitinases, and lipases, corroborated by additional factors and environmental conditions, endorse germination first, and then the penetration of the cuticle [22]. The subsequent growth of mycelium in the insect’s tissues leads to the host’s death and to the production of conidia on the surface of the corpse [23,24]. This is a key symptom indicating the presence and effect of this pathogen on dead insects. Secondary metabolites and the extracellular mucilaginous matrix, instead, further promote host invasion [17,25]. Due to its proven efficacy as a biocontrol agent of some groups of insect pests, *A. muscarius* IMI 268,317 (also known as Ve6, CBS 102.071, and ARSEF 5128) has been commercialised under the names Mycotal^®^ [26] to control whiteflies and thrips, and Verticillin^®^ for managing whiteflies, aphids, and mites [27].

Despite its lethal action against particular groups of pests, *A. muscarius* can live, in natural contexts, as an endophyte (e.g., on maple, pine, oak, laurel, and myrtle) or on crops (e.g., cucumber, sour cherry, and cabbage) [28]. The plethora of different hosts suggests a lack of host specialisation and indicates a potential permanent functional role in the natural ecosystem, possibly as a mechanism of defence [28]. This additional ecological function of *Akanthomyces* isolates was proven by direct attacks on powdery mildew, through chitinase production and mildew spore penetration [29], as well as by triggering plant defence responses and preventing damages caused by *Fusarium oxysporum* f.sp. *melonis* in melon [30,31].

Recently, *A. muscarius* has been found to be associated with hazelnut big buds infested by *P. avellanae* Nalepa, during a monitoring activity on a hazelnut orchard in the Viterbo area (Lazio, Italy; latitude 42°16′00.0″, longitude 12°17′00.0″, altitude 275 m) [32]. In winter–spring 2023, the same survey was repeated, assessing again the presence of *A. muscarius* [16] The persistent association between the fungus and the mite-infested big buds may suggest a possible involvement in big bud formation and/or defence against other hazelnut adversities.

Given the interesting features and characteristics of *A. muscarius*, and the limited knowledge about the Italian isolates, we hereby report the genomic characterisation of the AC3 isolate, firstly reported by Mazzaglia and colleagues [32]. We included its complete genome, feature annotation, and comparative analysis with other entomopathogenic isolates, with the aim to identify both common and species-specific virulence features. We believe that this is fundamental information that may open further doors to pest control strategies based on this entomopathogenic fungus.

## 2. Materials and Methods

### 2.1. Fungal Isolation and Culture Conditions

As reported by [32], in the framework of the European project PANTHEON, infested big buds were collected in a hazelnut field located in Viterbo province (Lazio, Italy; latitude 42°16′00.0″, longitude 12°17′00.0″, altitude 275 m). Living mites were picked with a needle from the galls and plated on Potato Dextrose Agar (PDA) plates amended with streptomycin (0.3 g L^−1^). The plates were incubated at 25 °C for up to 10 days and subsequently plates were monitored daily to assess microorganism growth. A morphological and molecular characterisation of *A. muscarius* AC3 was carried out by [32]. 

### 2.2. Total DNA Extraction 

Total genomic DNA was extracted from 10-day-old colonies of *A. muscarius* AC3 and grown on PDA. The CTAB-based protocol was the same as [33] with some modifications. Briefly, 500 mg of fresh mycelium was incubated with 750 µL of lysis buffer (2% CTAB, 0.02 M EDTA, 0.1 M Tris-HCl pH 8, and 1.2 M NaCl) and 10 µL of proteinase K (20 mg/mL) at 65 °C for one hour. One volume of chloroform–isoamyl alcohol (24:1) was then added to the solution, vortexed, and centrifuged at 5000 rpm for 10 min at room temperature. The aqueous phase was then incubated with 5 µL of RNAseA (10 mg/mL) and further extracted with a second round of chloroform–isoamyl alcohol. The aqueous phase was transferred into a new tube and the DNA was precipitated with 2/3 of cold isopropanol and 10% of Sodium Acetate, at 20 °C for 30 min. After 20 min of centrifugation at 5000 rpm, the pellet was washed with 70% ethanol and resuspended in 200 µL 65 °C preheated Tris-EDTA (TE). The DNA was quantified using the Invitrogen Qubit fluorometer (Thermo Fisher Scientific, Waltham, MA, USA) and used for library preparation.

### 2.3. ONT Library Preparation and Genome Sequencing

For Oxford Nanopore Technologies (ONT, Oxford, UK) sequencing, total DNA was first end-repaired using NEBNext Ultra II End Repair/dA Tailing Module (New England BioLabs, Ipswich, MA, USA) and libraries were prepared using the Ligation sequencing kit (SQK-LSK109). Sequencing was carried out on a MinION Mk1b device (ONT, Oxford, UK) using two R9.4.1 flow-cells (ONT). The base calling of the ONT long reads was carried out using Guppy within the MK1C device and the reads quality and sequencing statistics were evaluated using NanoPlot v. 1.30.1 [34]. An aliquot of the same DNA sample was sequenced at Eurofins Genomics (Eurofins Genomics GmbH, Konstanz, Germany) with the genome sequencer Illumina NovaSeq 6000 S2 (Illumina, San Diego, CA, USA) using the paired-end sequencing. The quality of the paired-end Illumina reads was evaluated using FastQC [35], before downstream analysis.

### 2.4. De Novo Hybrid Assembly

A backbone of the complete fungal genome was assembled by Canu v2.1.1 [36] using ONT long reads, with default parameters and by setting 40 Mb as the expected genome size. The assembly was further polished by Polca using the Illumina short reads [37]. Assembly quality statistics were evaluated using QUAST v5.0.2 [38], while the assembly completeness was evaluated with BUSCO v5.beta.1 [39], using *hypocreales_db10* as the ortholog lineage dataset which consists of a set of 4494 conserved proteins.

### 2.5. Structural and Functional Annotation

For structural annotation, RepeatModeler was initially used to create an internal database necessary to mask repetitive elements. MAKER pipeline v3.01.03 was then followed [40], setting the maximum intron size at 2500, single exon to 1, with Exonerate and tRNAscan-SE enabled to identify the tRNAs. Transcripts and proteins available from related species (*A. muscarius* Ve6, *Beauveria bassiana* ARSEF 2860, *C. fumosorosea* ARSEF 2679, *A. lecanii* UM487, *Cordycepes* sp. RAO2017, *C. javanica* IJ2G, and *C. militaris* CM01) were used as models by Est2Genome and Protein2Genome to identify intron and exon boundaries. SNAP and AUGUSTUS were used for an ab initio gene prediction in a second round of annotation. BLASTp with the latest version of SwissProt 2023_05 was used for functional annotation.

Functional groups belonging to the KEGG orthology were identified through BlastKOALA [41], while secondary metabolite Biosynthetic Gene Clusters (BGCs) were identified by AntiSMASH v6.0 [42]. dbCAN2 meta web server was used to identify the carbohydrate active enzymes (CAZymes) involved in carbohydrate metabolism [43], run with the implemented SignalP v4.0 for signal peptide identification [44].

### 2.6. AT-Rich Regions

In the fungal genome, AT-rich isochores are typically associated with the Repeat-Induced Point mutation (RIP), a defence mechanism that reduces the accumulation of repetitive DNA elements within the fungal genome and potentially maintains its stability. Thus, OcculterCut v1.1 was used to identify AT-rich regions, while the dinucleotide ratio related to possible RIP events was calculated by RIPCAL v.1.0 [45,46].

### 2.7. Comparative Genomics

For comparative genomics analysis, all the available annotated genomes belonging to the family Cordycipitaceae were downloaded from the NCBI Genome database (Table S1). For a more exhaustive analysis, the genome sequences of nine more *A. lecanii* isolates and eleven *A. dipterigenus* were downloaded and structurally annotated with MAKER as mentioned for the *A. muscarius* AC3 isolate. Orthologous proteins were identified by OrthoFinder v 2.5.5, and the results were further processed with the R package UpsetR v1.4 [47,48] within the R environment (v.4.2.3).

The species tree built by OrthoFinder was visualised in a dendrogram using FigTree v1.4.4 (available at http://tree.bio.ed.ac.uk/software/figtree/, accessed on 11 June 2024) and further edited with Inkscape v 0.92 (available at https://inkscape.org, accessed on 11 June 2024).

### 2.8. Pathogenicity- and Virulence-Related Genes

The entomopathogenic features summarised by Valero-Jiménez et al. [49] were downloaded from the NCBI Protein database and then searched through BLASTp on the orthogroup sequences identified by OrthoFinder. A graphical representation of the shared entomopathogenic-related features was carried out with the R package ComplexUpset v1.3.5 (available at https://zenodo.org/doi/10.5281/zenodo.3700590, accessed on 15 June 2024).

Additional pathogenicity- and virulence-related genes were searched through a BLASTp analysis using the latest Pathogen Host Interactions (PHI) database as reference [50]. Candidate apoplastic effectors were predicted among the proteins with signalP and without a transmembrane domain, with EffectorP v3 [51].

## 3. Results

### 3.1. Genome Sequencing and De Novo Assembly

The Illumina sequencing of the extracted HMW DNA produced high-quality 2 × 5 M reads (150 bp paired end) using a NovaSeq 6000 machine. Oxford Nanopore Technologies (ONT) sequencing on a Mk1C device using a R9.4.1 flow-cell generated 2.1 M reads (3.75 Gbp) with an N50 of 6534 and an average quality of 11.5. The draft genome sequence, assembled with Canu using only ONT long reads and polished with Polca using the short Illumina reads, consisted of 39 scaffolds, with a total length of 36.7 Mb, an N50 of 5.2 Mb, a GC content of 53.44%, and a coverage of 37.3× (Table 1). The genome was deposited on the NBCI Genome database under the accession number JAYJMN000000000.

### 3.2. Structural and Functional Annotation

**Figure 1:**
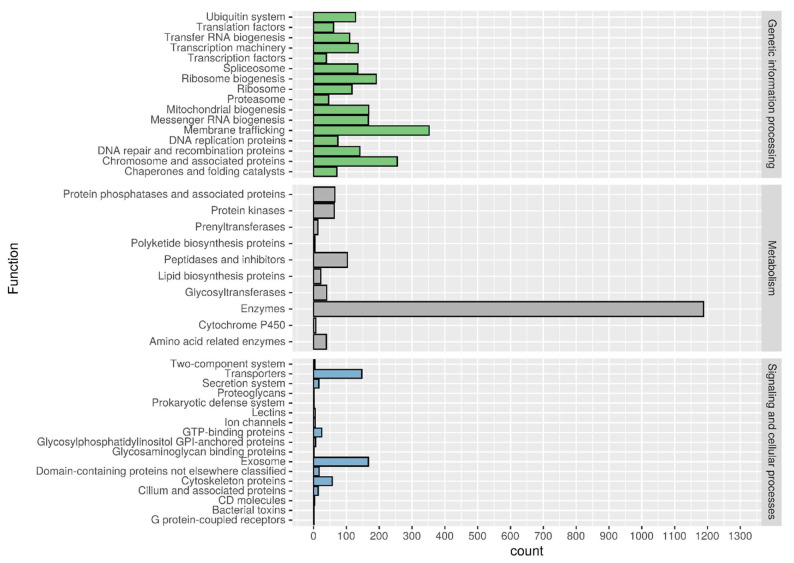
KEGG annotation and distribution among the different tree main classes: genetic information processing, metabolism and signalling, and cellular processes.

After two rounds of the MAKER2 annotation pipeline, a total of 10,901 putative genes and 133 tRNAs were annotated within the whole *A. muscarius* AC3 genome (Table 1). Functional annotations carried out through BLASTp and KEGG annotations carried out through BlastKoala show that the most abundant category is represented by the enzymes involved in metabolism (1188 putative proteins), followed by membrane trafficking (352), and chromosomes and associated proteins (255) from the genetic information processing cluster (Figure 1).

### 3.3. Repetitive Elements and AT-Rich Regions

**Figure 2:**
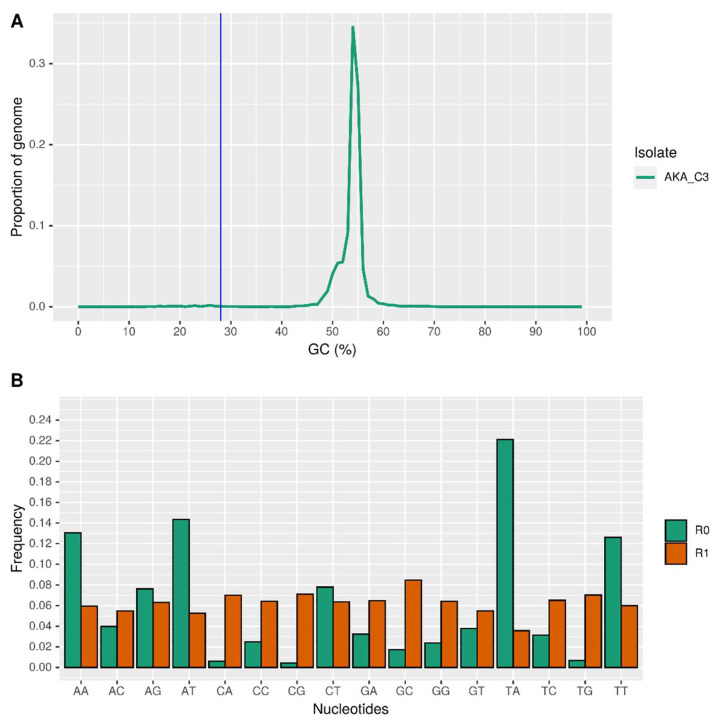
(**A**) Bimodal distribution of the GC content in the three genomes identified by Occultercut as enriched in AT (R0) and GC-equilibrated (R1) using a GC content threshold indicated by the vertical blue line. (**B**) Dinucleotide frequencies calculated by Occultercut in the R0 and R1 regions.

The assembled *A. muscarius* genome exhibited a bimodal distribution of GC content. A small portion (1.23%) of the genome was enriched in AT-rich regions (designated as R0 regions). These R0 regions had an average length of 5.97 kilobase pairs (kbp) and a peak GC content of 23.7%. Notably, no genes were found within these AT-rich regions. In contrast, the greatest portion of the genome (98.8%) consisted of GC-rich regions (designated as R1 regions). These R1 regions had a much larger average length of 343 kbp, a peak GC content of 54.4%, and a gene density of 300 genes per megabase pair (Mbp) (Figure 2A). One possible explanation for this bimodal GC-content distribution is the action of a fungal defence mechanism called Repeat-Induced Point mutation (RIP). RIP targets transposable elements (TEs), which are mobile segments of DNA, by methylating cytosines within these elements. These methylated cytosines can then be deaminated and converted to thymine [52]. This process would lead to an increase in AT content within the targeted TEs, potentially explaining the formation of the AT-rich R0 regions. To investigate the potential involvement of RIP in the formation of these AT-rich regions, we calculated the frequencies of all 16 possible dinucleotide combinations (AA, AT, etc.) in both R0 and R1 regions using the RIPCAL software. As expected, Figure 2B shows that the frequencies of dinucleotides containing A and T (AA, AT, TT, and TA) were significantly higher in the AT-rich R0 regions compared to the GC-rich R1 regions. However, when we calculated the RIP ratio values (TpA/ApT and (CpA + TpG)/(ApC + GpT)), we obtained values of 1.54 and 0.16, respectively. These values do not indicate the presence of active RIP in these regions according to established criteria. This finding was further supported by the absence of any “rid” alleles (specific DNA signatures associated with RIP) within the *A. muscarius* isolate, as confirmed by a BLASTn search against the sequences identified by [53].

### 3.4. Secondary Metabolites and Carbohydrate-Active Enzymes

**Figure 3:**
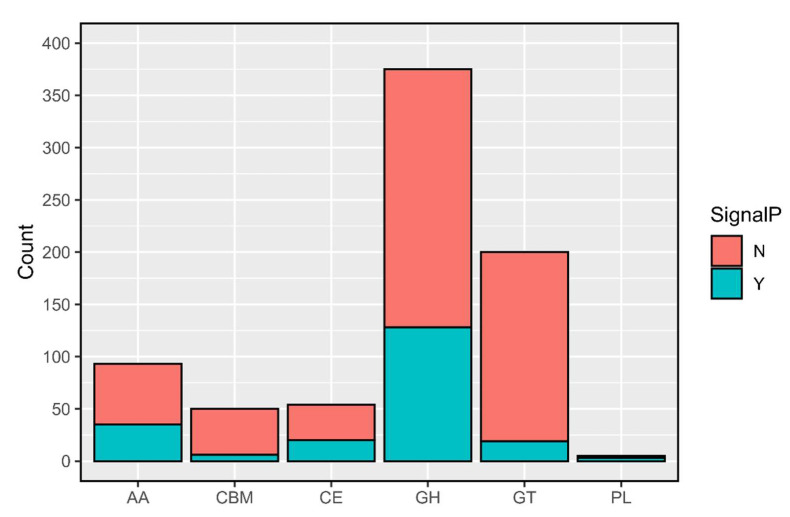
Count of the CAZyme families in the annotated features, with (Y) or without (N) signal peptides.

Antismash identified with 100% of identity 1,3,6,8-tetrahydroxynaphthalene polyketide (T1PKS), epichloenin A (NRPS), ε-Poly-L-lysine (NAPAA), choline (NRPS), fumosorinone (NRPS polyketide), and clavaric acid (Terpene), as well as a metachelin gene cluster (NRPS) with 75% of similarity (Table S2). The majority of the CAZymes (carbohydrate-active enzymes capable of synthesising or breaking down saccharides) identified using the three different tools on dbCAN web server (HMMER, DIAMOND and dbCAN, respectively) belonged to the family of glycoside hydrolases (GHs) without or with a signal peptide (247 and 128 features, respectively), the glycosyltransferases (GTs) without a signal peptide (181 features), and the CAZymes with auxiliary activity (AAs, 58 features without a signal peptide and 35 features with a signal peptide) (Figure 3).

### 3.5. Orthogroups Inference

**Figure 4:**
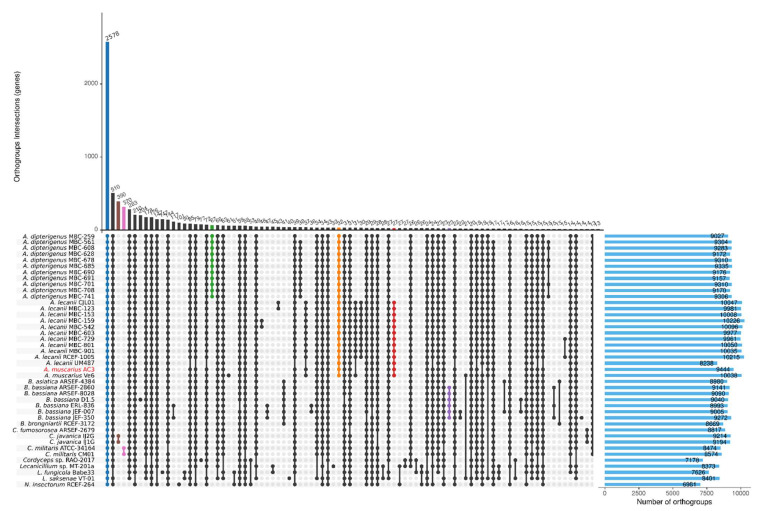
UpsetR plot showing the number of orthogroups identified by OrthoFinder and shared among the forty-two Cordycipitaceae isolates, indicated as dots connected by vertical bars. The horizontal blue bars on the right indicate the number of orthogroups per isolate. Coloured dots represent particular groups further described in the main text.

In a comparative analysis of the proteomes, OrthoFinder identified a total of 15,365 orthogroups, including 434,799 genes. Of these orthogroups, 2578 were shared among all the compared isolates, with 1367 of them as single copy, for a total of 217,021 genes (Figure 4). In particular, 390 orthogroups are specific for the *C. javanica* species, 320 for *C. militaris,* 67 orthogroups are shared among *A. dipterigenus* isolates, and 23 orthogroups are specific for the *B. bassiana* species. No characteristic orthogroups were found among the eleven *A. lecanii* isolates, but 32 orthogroups grouped the *Akanthomyces* genus (i.e., *A. dipterigenus, A. lecanii*, and *A. muscarius*). Interestingly, two orthogroups (ID OG0013532 and OG0013537) were found only in the two *A. muscarius* isolates AC3 and Ve6, both annotated as hypothetical proteins. Seven orthogroups were present only in the AC3 isolate: OG0012679 (three proteins, hypothetical protein HIM, putative transposase), OG0013522 (two proteins with no similarity found), OG0013523 (two proteins similar to DNAse I-like protein, putative RNA-dependent DNA polymerase), OG0013524 (two proteins with no similarity), OG0013525 (two proteins with no similarity), OG0013527 (two proteins similar to a transcription factor that binds to CRE motif and to Basic leucine zipper), and OG0013536 (two proteins similar to the beta-galactosidase lacA).

**Figure 5:**
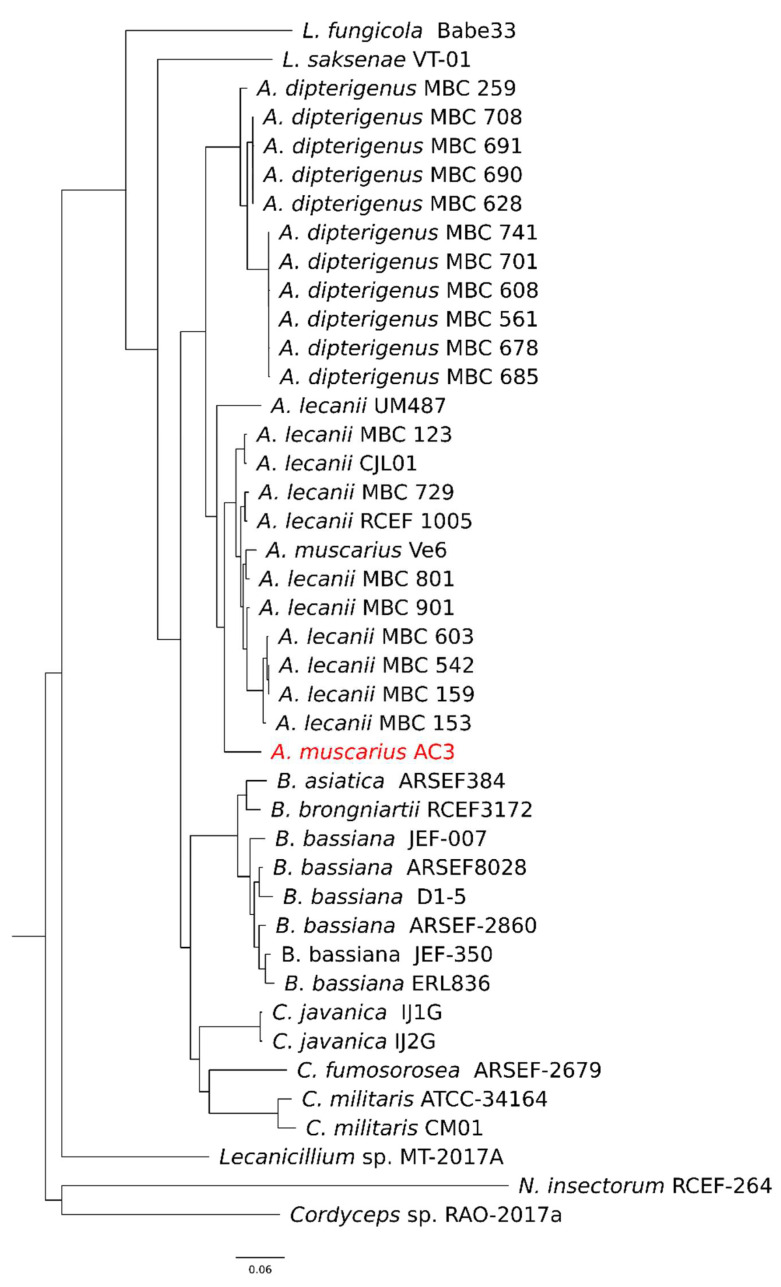
Species tree inferred among the 15,365 orthogroups identified by OrthoFinder rooted on *N. insectorum* RCEF-264 and *Cordyceps* sp. RAO-2017a. The *A. muscarius* AC3 isolate assembled in this work is indicated in red and clustered in the *Akanthomyces lecanii* and *A. musciarius* group.

In the species tree inferred by OrthoFinder, rooted on *N. insectorum* RCEF-264 and *Cordyceps* sp. RAO-2017a, distinct clusters were formed among *Akanthomyces*, *Beauveria*, and *Cordyceps* genera, with the two *A. muscarius* isolates grouped together with the *A. lecanii* isolates (Figure 5).

### 3.6. Entomopathogenic Fungus-Related Features

**Figure 6:**
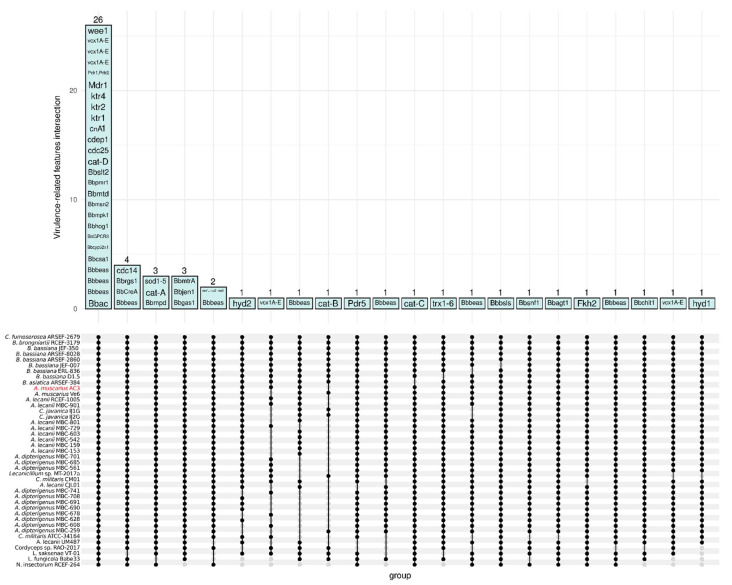
ComplexUpset plot showing the number of OrthoFinder orthogroups associated with entomopathogenic fungus-related features and shared among the forty-two Cordycipitaceae isolates, indicated as dots connected by vertical bars.

The thirty-eight *B. bassiana* genes involved in virulence and discussed by Valero-Jiménez et al. [49] were identified in 55 orthogroups inferred by OrthoFinder (Table 2). Among those, twenty-six orthogroups were shared among all the isolates under analysis, which include the Beauvericina toxin (bbbeas), cAMP signaling (Bbac), the neuronal calcium sensor (bbcsa), G-protein coupled receptor (BbGPCR3), MAP Kinase (BBhog1, Bbmpk1, Bbslt2), the predicted cytochrome P450 (Bbcyp52x1), the transcription factor (Bbmsn2), mannitol dehydrogenase (Bbmtd), the ATPase signaling (Bbpmr1), the catalase D (Cat-D), Cdk1 activity regulator (Cdc25, Wee1), the cuticle-degrading proteinase CDEP-1 (Cdep1), the Calcineuring-mediated phosphatase (CnA1), the a-1,2-mannosyltransferase (Ktr1, Ktr2, Ktr4), the ABC transporters (Mdr1, Pdr1, Pdr2), and the A-E calcium/proton exchanger (Vcx1). Four orthgroups (Bbbeas, BbCreA, Bbrgs1, and cdc14) were shared among all the isolates except *L. saksenae* VT-01, three orthgroups were shared among all except *L. fungicola* Babe33, and three other orthogroups among all except *N. insectorum* RCEF-264 (Figure 6).

### 3.7. Evaluation of the Secretome Features with Virulence Function

**Figure 7:**
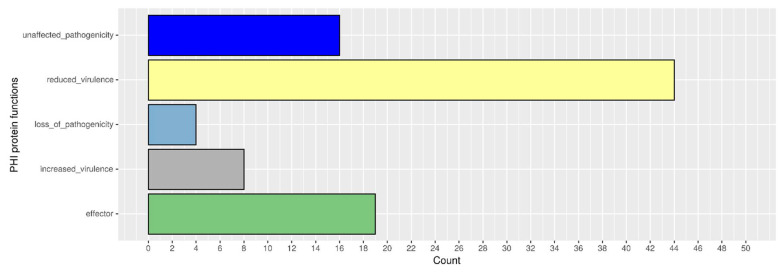
Putative pathogenicity-related (PHI-base) features identified in *A. muscarius* AC3 by BLASTp alignment towards the PHI database.

Effector proteins were predicted among those proteins with a signal peptide (1077 proteins) and without any transmembrane domain (888 proteins). EffectorP fungi 3.0 [51] identified 212 putative apoplastic effectors and 88 putative cytoplasmic effectors (Table S3). Of these 300 proteins, 91 had a match on the most recent PHI-base database (Figure 7), the majority of which (44 features) with reduced virulence (mutant phenotype), followed by the effector category (19 features), mostly represented by the Blys2 gene (PHI-7376) of *B. bassiana.* Furthermore, sixteen features were related to unaffected pathogenicity, eight features belonged to increased virulence, and four to the loss of pathogenicity category (Figure 7).

## 4. Discussion and Conclusions

The restrictive regulations on pesticide use currently applied in many European and worldwide countries [54] are endorsing studies on alternative pest control strategies [55]. This is the reason why, in this work, we reported and analysed the main feature of the genome of an isolate of *A. muscarius*, retrieved from a hazelnut field in Central Italy. An in-depth knowledge of the genetic aspects of entomopathogenic agents is crucial for developing targeted research that could lead to practical field applications. The natural diffusion of *A. muscarius* in different geographical areas worldwide leaves room for dedicated genetic and ecological studies that will further investigate the adaptability level of this organism. The identification of different isolates can surely be positive for potential applications, leading to new generation biopesticides that can be active on specific groups of pests and in different environmental conditions. The isolate from Central Italy can potentially extend the pool of commercial products such as Mycotal, once its efficacy is proven. Our work supported the preliminary step of this research, providing a piece of knowledge on an isolate naturally present in Central Italy. It is also worth pointing out that, to date, only one complete genome of *A. muscarius* is available on the NCBI database, specifically the Ve6 isolate [56]. In our study, both Illumina and Oxford Nanopore Technologies (ONT) sequencing were carried out to generate high-quality genomic data for *A. muscarius* AC3. The combination of these two methodologies has an established history and has been successfully used to assemble several fungal and bacterial genomes [33,57,58,59,60,61]. The draft genome, assembled and polished using Canu and Polca, consists of 39 scaffolds totalling 36.7 Mb and 10,901 putative genes, in line with the *Akanthomyces* genus. Functional annotations revealed that the most abundant protein categories are metabolic enzymes (1188) and membrane trafficking proteins (352), which could be involved in the firsts step of host colonisation [49]. The assembled *A. muscarius* genome showed a bimodal GC content distribution, with 1.23% of the genome being AT-rich (R0 regions) and 98.8% GC-rich (R1 regions). The R0 regions, averaging 5.97 kbp with a peak GC content of 23.7%, contained no genes. In contrast, the R1 regions, averaging 343 kbp with a peak GC content of 54.4%, had a gene density of 300 genes per Mbp. The bimodal distribution is potentially due to the fungal defence mechanism called Repeat-Induced Point mutation (RIP), which targets transposable elements (TEs). However, analysis using RIPCAL software and RIP ratio values did not confirm active RIP in these regions, a finding supported by the absence of “rid” alleles in the genome.

The comparative genomics carried out on entomopathogenic fungi in the Cordycipitaceae family highlighted important genetic information of our *A. muscarius* isolate compared to strains previously described in the literature. Additionally, the genome of *A. muscarius* contains several genes responsible for virulence regulation that have been previously identified in other entomopathogenic species, such as *B. bassiana* [49]. This pool of genes is responsible for the infection process over time, since they regulate the crucial steps ranging from the initial contact to the emergence from the host after its death [50].

In more detail, our *A. muscarius* isolate contains the genes *Bbhog1* and *Bbmpk1* that regulate the first step of the infection process, namely, host adhesion [62]. Those genes are responsible for the adhesion on the external surface of the cuticular region of the host insect and control part of the morphological development process, such as the germination of conidia and the secretion of enzymes for haemocoel penetration, mostly proteases [63].

The genome also showed a set of genes known for their role in the infection stages that take place inside the body of the host insect. The presence of some of them, considered to be crucial for the fungus life cycle, regulate important steps as follows: once inside the haemocoel, the fungus regulates its interaction with the hemolymph through genes such as *Bbsnf1*, that controls the development of the blastospores, while genes such as *Bbbeas* guarantee the production of toxins that damage the internal part of the host insect [64]. The perforation of the cuticle that leads to the host’s death, the emergence, and the sporulation that spreads the pathogen towards other hosts, is instead regulated by genes such as *Bbgas1*, *Bbjen1*, or *Bbpmr1* [65,66,67].

The presence of genes that endorse entomopathogenic activity within the genome of the isolate of *A. muscarius* investigated in this study is an in silico proof that opens the door to laboratory trials on the entomopathogenic activity on selected host insect species. As a next step, in fact, the efficacy and the gene expression during key infection stages should be assessed. Identifying suitable host species, instead, can be achieved through extensive surveys, followed by the isolation of the microbiome associated with the insect specimens collected. Possibly, the starting point should be symptomatic samples or groups of insects, especially those known to be infected by *A. muscarius*.

This would be a potential ally to control pest infestations in hazelnut orchards in a highly productive area such as Central Italy. The natural presence of the fungus even in intensively cultivated areas is a positive aspect that deserves future investigations to understand the main insect hosts and how agronomic practices affect the biology of this natural enemy. Another interesting aspect that is worthy of discussion is the potential relationship with the bud mite *P. avellanae*, from which we isolated *A. muscarius*. To date, there is limited knowledge on the biology and the ecology of this pest, as well as most of the mechanisms behind big bud formation [68]. Given that AC3 was isolated from galls associated with *P. avellanae*, further studies may be directed towards a better understanding of the relationship between the two organisms.

Overall, the genetic information provided with this study poses many questions about the biology and ecology of *A. muscarius*, as well as its potential role as a biological controller of many insect pest species infesting crops and orchards. Besides the applications in agriculture, in fact, there might be great benefits to reduce the infestations in particular environments such as urban parks or natural areas, where conventional treatments are not allowed by law. Our study is fully inserted in this framework, laying the foundations for additional tools to be applied in sustainable pest control.

## Figures and Tables

**Table 1:** Summary statistics of the *A. muscarius* AC3 isolate genome.

Summary Statistics	*A. muscarius* AC3
**Assembly Statistics**	
# contigs	39
Largest contig	7,812,308
Total length	36,760,018
N50	3,276,623
L50	3
GC (%)	53.44
Mismatches	
# N’s per 100 kbp	0
# N’s	0
**BUSCO completeness**	
BUSCO completeness	99.5%
Complete	4472
Single-copy	4460
Duplicated	12
Fragmented	3
Missing	19
**Protein coding genes**	
Protein coding genes	10,901
Predicted tRNAs	133
Proteins with signal peptide	1077
Predicted effector proteins	300
Predicted cytoplasmic effectors	88
Predicted apoplastic effectors	212

**Table 2:** List of entomopathogenic features discussed by [49] and here identified in the orthogroups.

Orthogroup	Gene_Name	Function	Accession Number
OG0004073	*Bbac*	cAMP signaling	EJP69082.1
OG0005551	*Bbagt1*	α-glucose transporter	AFN69126.1
OG0000595, OG0000996, OG0001347, OG0001550, OG0003256, OG0004772, OG0004773, OG0006500, OG0007415	*Bbbeas*	Beauvericina toxin	ACI30659.1, ACI30658.1, ACI30657.1 ACI30656.1, ACI30655.1.ACI30654.1 ACI30653.1.ACI30652.1, ACI30651.1
OG0005080	*Bbbsls*	Bassianolide synthetase	ACR78148.1, ACR78134.1
OG0006326	*Bbchit1*	chitinase	AAN41259.1
OG0002032	*BbCreA*	CreA Aspergillus	AXG50936.1
OG0002053	*Bbcsa1*	Neuronal calcium sensor	AFG25510.1
OG0000010	*Bbcyp52x1*	Predicted_cytochrome_P450	ADK36660.1
OG0001161	*Bbgas1*	Cell-wall anchored enzyme	ADP22300.1
OG0000852	*BbGPCR3*	G-protein coupled receptor	XP_008594187.1
OG0001208	*Bbhog1*	MAP kinase	AAS77871.1
OG0002187	*Bbjen1*	Carboxylate transporter	AAO33825.1
OG0003168	*Bbmpd*	Mannitol-1-phosphate-dehydrogenase	ACU32784.1
OG0003410	*Bbmpk1*	MAP kinase	AAQ24633.1
OG0001461	*Bbmsn2*	Transcription factor	EJP70102.1
OG0001442	*Bbmtd*	Mannitol dehydrogenase	ADV16370.1
OG0000103	*BbmtrA*	Putative methyltransferase	EJP69472.1
OG0000771	*Bbpmr1*	ATPase, signaling	AAY40175
OG0005594	*Bbrgs1*	G-protein signaling	ABL61517.1
OG0000669	*Bbslt2*	MAP kinase	AEU60018.1
OG0005917	*Bbsnf1*	Protein kinase	XP_008597580.1
OG0003206, OG0009475, OG0000753, OG0000172	*cat-A*, *cat-B*, *cat-C*, *cat-D*	Catalase A, B C and catalase D	JX050138.1, JX050139.1.JX050141.1, JX050142, JX050140
OG0005553	*cdc14*	Dual specificity phosphatase	EJP63157.1
OG0004028	*cdc25*	Regulates Cdk1 activity	EJP68939
OG0000004	*cdep1*	Cuticle-degrading_proteinase_CDEP-1	AAK70804.1
OG0000261	*CnA1*, *CnA2*, *CnB*, *Crz1*	Calcineuring-mediated phosphatase	XP_008596022.1
OG0000461	*Fkh2*	Transcription factor	EJP62625.1
OG0007102, OG0006455	*Hyd1*, *hyd2*	Hydrophobin, hydrophobin	ABO38181
			ABP58683.1
OG0002106, OG0002128, OG0002169	*ktr1,* *ktr2*, *ktr4*	a-1,2-mannosyltransferase	EJP63998.1, EJP62226.1, EJP69973.1
OG0001805	*Mdr1*	ABC transporters	XP_008595100, XP_008602098.1, XP_008600979.1, XP_008594344.1,
OG0000167	*Pdr1*, *Pdr2*	ABC transporters	XP_008595100, XP_008602098.1, XP_008600979.1, XP_008594344.1,
OG0001832	*Pdr5*	ABC transporters	XP_008595100, XP_008602098.1, XP_008600979.1, XP_008594344.1,
OG0006835	*ras1*, *ras2*, *ras3*	GTPase, signal transduction	EJP70406.1
OG0005188	*sod1-5*	Superoxide dismutases	EJP67024.1
OG0006040	*trx1-6*	Thioredoxin antioxidants	ACT21200.1
OG0002437, OG0003500, OG0004231, OG0004459, OG0008389	*vcx1A-E*	Calcium/proton exchanger	EJP69434.1, EJP63266.1, EJP67299.1, EJP69455.1, EJP69457.1
OG0001590	*wee1*	Regulates Cdk1 activity	EJP62459

## Data Availability

The genome assembled in this work is available on the NCBI Genome database under the accession number JAYJMN000000000.

## Supplementary Materials

The following supporting information can be downloaded at: https://www.mdpi.com/article/10.3390/genes15080993/s1, Table S1: List of all the NCBI available annotated genomes belonging to the family Cordycipitaceae used for comparative genomics. Table S2: List of secondary metabolite Biosynthetic Gene Clusters (BGCs) identified by Antismash; Table S3: Putative apoplastic and cytoplasmic effectors identified by EffectorP among the annotated features carrying a signal peptide and without a transmembrane domain.
